# Clusters of long COVID among patients hospitalized for COVID-19 in New York City

**DOI:** 10.1186/s12889-024-19379-9

**Published:** 2024-07-25

**Authors:** Sara Venkatraman, Jesus Maria Gomez Salinero, Adina Scheinfeld, Sean Houghton, David Redmond, Monika Safford, Mangala Rajan

**Affiliations:** 1 Division of General Internal Medicine, Department of Medicine, Weill Cornell Medicine, 420 E 70th street LH 348, New York, NY USA; 2 Division of Regenerative Medicine, Department of Medicine, Hartman Institute for Therapeutic Organ Regeneration, Weill Cornell Medicine, New York, NY USA; 3 Lewis-Sigler Institute for Integrative Genomics, Princeton University, Princeton, NJ USA; 4 Department of Statistics and Data Science, Cornell University, Ithaca, NY USA

**Keywords:** COVID-19, Long-COVID, Cluster analysis, Social isolation, Loneliness

## Abstract

**Background:**

Recent studies have demonstrated that individuals hospitalized due to COVID-19 can be affected by “long-COVID” symptoms for as long as one year after discharge.

**Objectives:**

Our study objective is to identify data-driven clusters of patients using a novel, unsupervised machine learning technique.

**Methods:**

The study uses data from 437 patients hospitalized in New York City between March 3rd and May 15th of 2020. The data used was abstracted from medical records and collected from a follow-up survey for up to one-year post-hospitalization. Hospitalization data included demographics, comorbidities, and in-hospital complications. The survey collected long-COVID symptoms, and information on general health, social isolation, and loneliness. To perform the analysis, we created a graph by projecting the data onto eight principal components (PCs) and running the K-nearest neighbors algorithm. We then used Louvain’s algorithm to partition this graph into non-overlapping clusters.

**Results:**

The cluster analysis produced four clusters with distinct health and social connectivity patterns. The first cluster (*n* = 141) consisted of patients with both long-COVID neurological symptoms (74%) and social isolation/loneliness. The second cluster (*n* = 137) consisted of healthy patients who were also more socially connected and not lonely. The third cluster (*n* = 96) contained patients with neurological symptoms who were socially connected but lonely, and the fourth cluster (*n* = 63) consisted entirely of patients who had traumatic COVID hospitalization, were intubated, suffered symptoms, but were socially connected and experienced recovery.

**Conclusion:**

The cluster analysis identified social isolation and loneliness as important features associated with long-COVID symptoms and recovery after hospitalization. It also confirms that social isolation and loneliness, though connected, are not necessarily the same. Physicians need to be aware of how social characteristics relate to long-COVID and patient’s ability to cope with the resulting symptoms.

**Supplementary Information:**

The online version contains supplementary material available at 10.1186/s12889-024-19379-9.

## Introduction

Patients who experience COVID-19 can present with symptoms after recovering from acute infection. This condition, called “long-COVID” or “post-acute sequalae of COVID-19 (PASC),” is a collection of symptoms that affect the daily functioning of patients up to 1 year after hospitalization. [1] Given the heterogeneity of symptoms reported, the World Health Organization released a definition describing the condition as symptoms lasting for over 2 months after acute COVID-19 infection, that cannot be directly attributed to other conditions that that patient may have. [2]

Starting in March 2020, New York City (NYC) was the epicenter of the COVID-19 pandemic. Several studies reported on patients who were hospitalized in NYC during this time. [3–6] Further, studies have also reported on the post-acute presentation of symptoms among patients hospitalized with COVID-19 in the area. [7–10] This early exposure to COVID-19 and subsequent quick spread throughout the city occurred while public health authorities were implementing interventions in terms of both social distancing and vaccines. Notably, the city-mandated closure of schools beginning on March 16, 2020 was the first intervention meant to address the growing severity of outbreaks across the city. [11] These initial school closures were followed by broad closures on March 22nd 2020. [11]. In this study we wish to characterize long-COVID among one cohort of patients hospitalized for COVID-19 in New York City prior to the availability of vaccines.

Cluster analysis is an unsupervised classification technique typically used to characterize patient phenotypes for a given condition. Among the initial hospitalizations in NYC, one recent study has used cluster analysis methods to categorize phenotypes, [9] and another has used regression based analyses to identify the role of several life stressors in post-acute symptoms. However, these analyses did not utilize data from the hospitalization for acute COVID-19 [10].

The objective of this study was to compile and analyze all data available from both the initial hospitalization of COVID-19 patients [3] and the follow-up communication in which patients reported long COVID symptoms. [1] This allowed us to identify data-driven clusters that clearly classified patient phenotypes and evaluate the importance of specific features within each patient cluster. We hypothesized that social isolation, defined as “a state in which the individual lacks a sense of belonging socially, lacks engagement with others, has a minimal number of social contacts and they are deficient in fulfilling and quality relationships” would be an important feature in at least some of the clusters. [12] In addition, we also explored “loneliness”, a related construct. Loneliness is normally understood as a “.discrepancy between a person’s preferred and actual level of social contact”. As compared to social isolation, which objectively measures the level of a person’s social contact, loneliness is a more subjective measure [13]. Both problems are significant among older adults and are known to be associated with poorer health outcomes. [14]

## Methods

The details of the study setting and the data sources utilized were published in a prior analysis. [1] In short, this study analyzes data collected from patients who were hospitalized in New York-Presbyterian (NYP)/Weill Cornell Medical Center (WCMC) or NYP/Lower Manhattan Hospital (LMH) between March 3rd and May 15th, 2020 who also responded to a follow-up phone survey between nine months and one year following the hospitalization. We used a wide range of data elements from the original COVID-19 hospitalization and information collected over the phone.

### Features used

From the hospitalization, we used information on admission status (whether from home or another setting), whether the patient was a health worker, age, gender, Race, BMI, smoking status, hypoxia at initial presentation, and comorbidity status (hypertension, diabetes, coronary artery disease, cancer, liver disease (Hepatitis, cirrhosis), lung disease, renal disease (CKD, ESRD) and immunocompromised status). We then added elements from hospitalization including intubation status, complications (acute kidney injury, dialysis, venous thromboembolism), and discharge disposition (death or discharge disposition).

During the follow-up phone call survey, respondents were asked about their long-COVID symptoms and health status (SF-1), rated from excellent to poor, and about any changes in health status from one year ago. The survey also asked about functional ability for daily activities, and social isolation and loneliness. Social isolation was measured using the Lubben Social Network Scale–6 (LSNS-6), a six-item, self-reported, scale to assess social isolation in older adults (range: 0 to 30, with higher scores indicating greater connectedness). Loneliness was measured using the UCLA 3-item Loneliness scale (range: 0 to 9, with higher scores indicating greater loneliness). [15, 16] The complete list of features selected for the analysis is listed in Supplementary Table 1.

### Statistical methods

In the cluster analysis, we included a comprehensive list of features including the 6 LSNS features, the Lubben composite score, the 3 UCLA Loneliness features, and the UCLA composite score. To perform the analysis, we utilized a graphical clustering technique, typically used for gene classification, that detects community networks using an optimization method called modularity. The modularity optimization technique maximizes the difference between the actual and expected number of edges in a community using Louvain’s algorithm.

We chose this approach as it allowed us to use all available data from the initial hospitalization and the follow-up phone call, which avoided the need for data selection. Using all patient features, the algorithm formed clusters of patient communities with strong common features (nodes) within clusters and weak connections between clusters.

We operationalized this by using the R software package Seurat (v 3.2.3). [17] The first step in the pre-processing was to normalize all variables included in the analysis. Following normalization, we conducted a principal component (PC) analysis and verified the amount of variance explained by the top principal components. Visual inspection of PCs above 8 showed a more homogeneous expression of the composing characteristics with no clear separation. Hence, 8 PCs were retained for downstream analysis.

The clustering process was initiated by the creation of a graph using the K-Nearest Neighbor with Euclidean distance in the PC space with the objective of “finding neighbors.” The clustering was further refined based on shared overlap of features between neighborhoods. The final cluster solution was created using Louvain’s algorithm with a resolution of 0.5.

We have described and named the clusters and checked for the significance of features using the log 2-fold change (Log2 FC) metric. The Log2 FC metric measures the increase in the expression of a specific feature and is expressed in log terms. A positive score implies higher expression of that feature in that cluster as compared with the expression of that feature in other clusters.

## Results

**Fig. 1:**
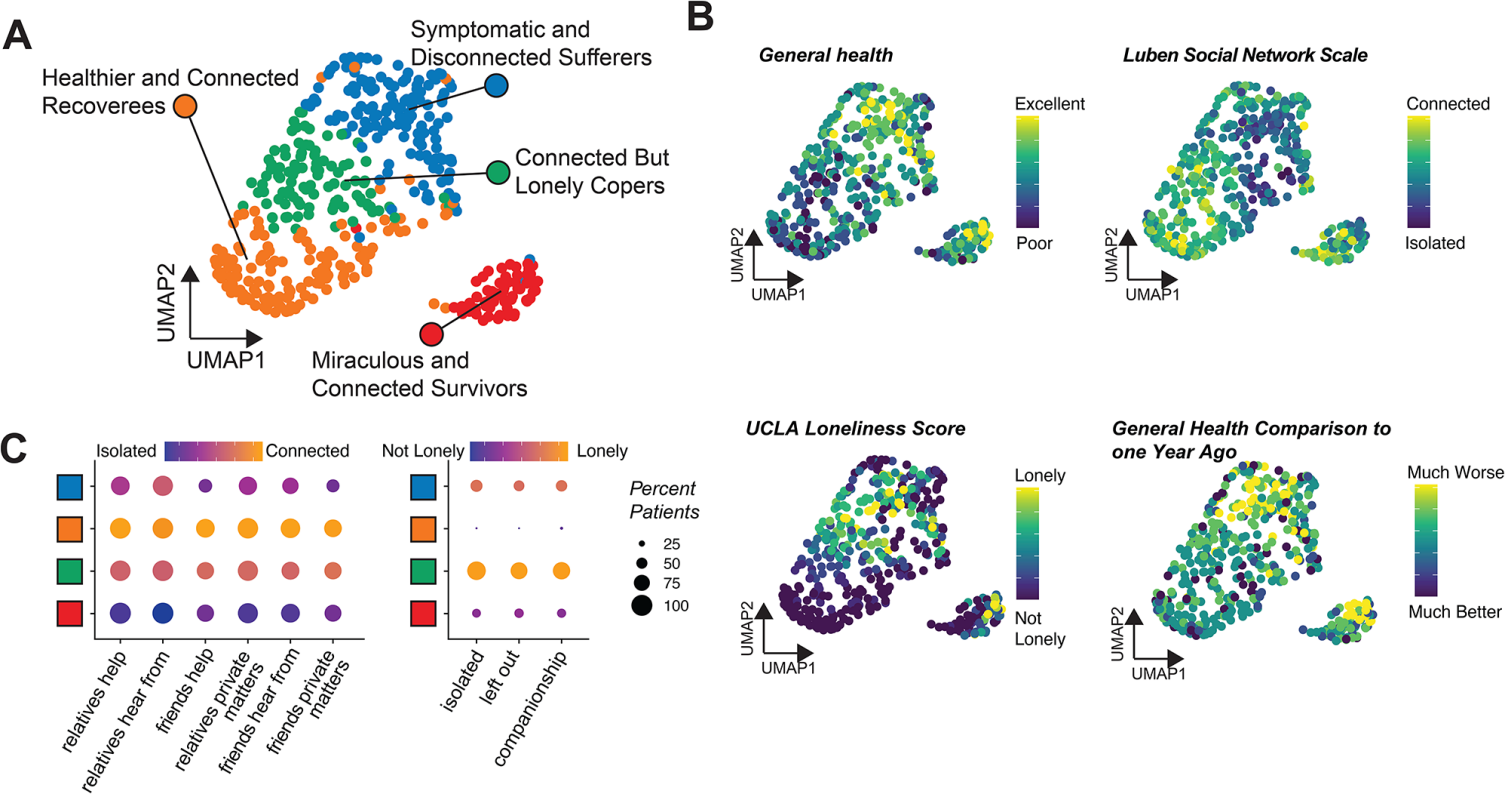
Visual summary of patient clusters. (**A**) and (**B**) display two-dimensional projections of the 437 patients obtained via UMAP, a dimensionality reduction technique that can be used for visualizing high-dimensional data. (**A**) colors patients by cluster (cluster 0 in blue; cluster 1 in orange; cluster 2 in green; cluster 3 in red). (**B**) colors patients by values of four of their characteristics: self-assessment of general health, Lubben Social Network Scale, UCLA Loneliness scale, and self-assessment of general health compared to one year ago. (**C**) displays how patients in each cluster responded to each of the six questions included in the Lubben Social Network Scale and the three questions included in the UCLA Loneliness Scale

Using complete data from 437 patients, our analysis produced four clusters comprised of 141, 137, 96, and 63 patients, which we refer to as clusters 0, 1, 2, and 3, respectively. In Fig. 1, we plot the projections of these patients into their first two UMAP components and observe that the four clusters follow distinct visual groupings. In Supplementary Table 1, we summarize these clusters of patients in terms of their long COVID symptoms, general health, and social isolation and loneliness. In Supplementary Table 2, we present the comorbidities, health characteristics, and in-hospital complications related to the patients’ original COVID hospitalization.

Patients in cluster 0 had more symptoms of long COVID than patients in other clusters and were also more socially isolated, with an average Lubben score of 12 and a UCLA Loneliness score of 3. The total Lubben score as well as all 6 individual components representing poor social connectedness were significantly expressed within this cluster. Patients in this cluster also experienced neurological symptoms such as brain fog and difficulty concentrating or sleeping (74%), respiratory symptoms such as coughing or shortness of breath (54%), and chest pain (15%). They also experienced poor general health with 59% reporting fair or poor health. These patients also had greater challenges in daily activity as compared with patients in the other clusters, including moderate activities (79%), climbing stairs (93%), bending (77%), and walking one mile (93%) or several blocks (84%). We called this cluster “Symptomatic and Disconnected Sufferers.”

Cluster 1 consisted of healthier patients who were also more socially connected and less lonely. These patients had a high Lubben score (19) and a low loneliness score (0). The total UCLA Loneliness score along with each of the three individual components representing minimal loneliness were significantly expressed in this cluster. Patients in this cluster had lower levels of adverse symptoms associated with concentrating or sleeping (23%), respiratory symptoms such as coughing (13%) and chest pain (2.9%). These patients were predominantly male (65%) and were in good general health, with 87% reporting that their health was good, very good, or excellent, much higher than the other groups (41.6%, 81%, and 58.9% for clusters 0, 2, and 3, respectively). More than 4/5th (82%) also reported that their general health was about the same, somewhat better, or much better than it was one year ago, again the highest proportion among the groups (40%, 63.5%, and 48.8% for clusters 0, 2, and 3, respectively). We called this cluster “Healthier and Connected Recoverees.”

Patients in cluster 2 had a similarly high average Lubben score of 18 but also a higher loneliness score of 4, thus demonstrating that social connectivity is not mutually exclusive from loneliness. The UCLA Loneliness score, along with the three individual components representing extreme loneliness were the top 5 features significantly expressed in this cluster. About 59% of these patients reported feeling isolated from others sometimes or often, and 58% reported feeling that they lacked companionship. They reported moderate levels of neurological symptoms (54%), respiratory symptoms (23%), and chest pain (7.3%). We labeled this group “Connected but Lonely Copers.”

Cluster 3, which appears separated from all other clusters in Fig. 1, consisted entirely of intubated patients. These patients, however, were socially connected and not lonely, with an average Lubben score of 20 and loneliness score of 1. They were primarily male (74.6%) and had the highest length of hospital stay with a median of 40 days (IQR 24–67) as compared with a median of four days in each of the other three clusters. Cluster 3 also consisted of patients with more severe in-hospital symptoms, including ARDS (47%) and hypoxia (78%) and had complications including AKI (63%), septic shock (25%), ventricular pneumonia (33%), VTE (75%), new onset arrythmia (25%), and dialysis (25%). Patients in this cluster experienced long COVID neurological symptoms (60%), respiratory symptoms (44%) and numbness in hands or feet (60%), and were evenly divided between those who had trouble with physical activities and those who did not. Interestingly, 41% reported fair or poor general health, a much lower proportion than in cluster 0, the other symptomatic cluster. We called this cluster “Miraculous and Connected Survivors.”

## Discussion

Using a combination of in-hospital and post-hospital follow-up data from the initial wave of COVID patients in NYC, our study demonstrates that, after consideration of all other features, there exists a relationship between social connectivity, loneliness, and long-COVID symptoms among adult patients hospitalized for the disease. The study identifies Cluster 0, “Symptomatic and Disconnected Sufferers,” those who deal with the worst effects of long COVID along with social isolation; Cluster 1, “Healthier and Connected Recoverees,” those who exhibit lower levels of symptoms and healthy social connectedness; Cluster 2, “Connected But Lonely Copers,” those who exemplify that social isolation and loneliness, though connected, are not necessarily the same; and finally Cluster 3, “Miraculous and Connected Survivors,” those who underwent stressful hospitalization but show remarkable recovery and healthy social connectedness.

Our study is unique in that it utilized a comprehensive list of features from both the original hospitalization and the follow-up survey to reveal clusters that highlight the significance of social isolation and loneliness. Aspects of these features were significantly expressed in 3 of the 4 clusters, and within the “Connected But Lonely Copers,” loneliness represented the most important feature.

Understanding social isolation and loneliness is becoming more important in healthcare, as it is associated with poor health outcomes, especially in older adults [13, 14, 18, 19]. However, the impact of these social conditions on long COVID is still relatively unclear. One could posit that support from peers could help patients cope with the long COVID symptoms and situations of stress surrounding the condition [20]. Most importantly, physicians treating these patients should probe more deeply into social characteristics to understand how they will cope with this kind of illness long-term.

Another important finding of our study is the differences in the expression of social connectedness and loneliness. Our results highlight the differences between these measures and reveal clusters that discriminate between the two. One cluster specifically displays higher social connectedness and high loneliness (“Connected but Lonely Copers”), indicating that though correlated, they are distinct constructs. There are studies showing that social connectedness is associated with better mental health and ability to cope with stressors, [20] and that both poor social connectedness and loneliness are independently associated with poor outcomes in adults [18] or that one can modify the impact of the other. [19] Further study on how these two social constructs affect long COVID symptoms and recovery is essential.

Our study used a novel clustering technique, typically applied to genetics, to classify hospitalized patients using an extensive list of features from the original COVID hospitalization and the follow-up survey. Another study from the New York area performed an unsupervised cluster analysis and identified three symptom and three therapeutic clusters which were mapped to classify the different symptoms and therapies used. [9] However, this study utilized only the symptoms and therapies to feed the cluster analysis and did not demonstrate any differences by social isolation. A different study used data from the RECOVER electronic health record database in the New York area to characterize the physical conditions and symptoms more likely to occur in patients with COVID compared to those without COVID, but they did not include any social measures in their analysis [21].

The importance of social isolation and loneliness in healthcare is driven by their association with adverse downstream outcomes for patients. For instance, loneliness can impact healthcare access. Prior studies show that loneliness brought about by the loss of a loved one or partner can disrupt access to care, especially if that person was the main source of transportation or a driver [22]. On the other hand, one study showed that older women who are lonely tend to have increased healthcare use, underscoring the complexity of this association [23]. Further, social Isolation and loneliness can impact nutrition and food intake. However, the direction of this relationship is unclear, and still being studied [24]. 

Our study comes with a few limitations. Firstly, the data were gathered prior to specific definitions for “post-acute” being developed [25, 26] thus we are using a collection of long-COVID symptoms that were reported, but not yet part of any consensus or definition. The post-hospitalization follow-up survey data was also collected as a one-time survey with a response rate of ~ 50%. Despite these limitations, this study demonstrates patterns of social isolation and loneliness that are present in patients after COVID-19 hospitalization. Future studies could examine post-acute symptoms over waves of COVID-19 defined by different variants of the disease, especially those that occurred after mobility restrictions were eased in urban environments, social interaction began returning and vaccines were widely available.

In conclusion, the cluster analysis identified social isolation and loneliness as important features associated with long-COVID symptoms and recovery after hospitalization. It also confirms that social isolation and loneliness, though connected, are not necessarily the same. Physicians need to be aware of how social characteristics relate to long-COVID and patient’s ability to cope with the resulting symptoms.

### Electronic supplementary material

Below is the link to the electronic supplementary material.

Supplementary Material 1

## Data Availability

The datasets generated and/or analyzed during the current study are not publicly available due to the proprietary nature of the data but are available from the corresponding author on reasonable request.
