# Supplementary material for: Clusters of long COVID among patients hospitalized for COVID-19 in New York City
Source: BMC Public Health. 2024 Jul 25;24:1994. doi: 10.1186/s12889-024-19379-9 (PMC11282800; doi:10.1186/s12889-024-19379-9)
Supplement: Supplementary file 1 — Supplementary Material 1 [file 12889_2024_19379_MOESM1_ESM.docx]

Supplementary Table 1 – Additional Cluster Profiles – Demographics, Long COVID Symptoms and Social Isolation and Loneliness – 9 months to 1 year after Acute COVID Illness

| **Characteristic (%)** | **Cluster 0** (141) | **Cluster 1** (137) | **Cluster 2** (96) | **Cluster 3** (63) | **Total** (437) |
| --- | --- | --- | --- | --- | --- |
| **DEMOGRAPHICS** |  |  |  |  |  |
| **Age category** |  |  |  |  |  |
| *Less than 25* | 0.0 | 0.7 | 1.0 | 1.6 | 0.7 |
| *26 - 40* | 7.1 | 10.0 | 16.0 | 7.9 | 10.0 |
| *41 - 55* | 14.0 | 28.0 | 23.0 | 22.0 | 22.0 |
| *56 - 70* | 45.0 | 39.0 | 29.0 | 46.0 | 40.0 |
| *71 - 85* | 21.0 | 21.0 | 24.0 | 21.0 | 22.0 |
| *86+* | 13.0 | 1.5 | 7.3 | 1.6 | 6.4 |
| **Female** | 54.0 | 35.0 | 48.0 | 25.0 | 43.0 |
| **Race/Ethnicity** |  |  |  |  |  |
| *Hispanic* | 32.0 | 18.0 | 21.0 | 29.0 | 24.0 |
| *African-American* | 17.0 | 8.8 | 12.0 | 7.9 | 12.0 |
| *Asian* | 12.0 | 16.0 | 15.0 | 21.0 | 15.0 |
| *White* | 24.0 | 31.0 | 34.0 | 32.0 | 30.0 |
| *Other* | 15.0 | 26.0 | 18.0 | 11.0 | 19.0 |
| **LONG COVID SYMPTOMS (% Few or several times a week)** | | |  |  |  |
| **Neurological Symptoms** – Brain Fog, Trouble Concentrating, Insomnia | 74.0 | 23.0 | 54.0 | 60.0 | 52.0 |
| **Respiratory Symptoms** - Cough, Shortness of Breath | 54.0 | 13.0 | 23.0 | 44.0 | 33.0 |
| **Other Symptoms:** |  |  |  |  |  |
| Chest Pain | 15.0 | 2.9 | 7.3 | 9.5 | 8.7 |
| Loss of Taste/Smell | 11.0 | 4.4 | 1.0 | 14.0 | 7.3 |
| Numbness/Weakness in hand, arm, leg, or foot | 43.0 | 12.0 | 14.0 | 60.0 | 30.0 |
| **General Health** |  |  |  |  |  |
| Excellent | 2.1 | 20.0 | 11.0 | 7.9 | 11.0 |
| Very good | 8.5 | 36.0 | 25.0 | 19.0 | 22.0 |
| Good | 31.0 | 31.0 | 45.0 | 32.0 | 34.0 |
| Fair | 43.0 | 11.0 | 17.0 | 25.0 | 24.0 |
| Poor | 16.0 | 0.7 | 2.1 | 16.0 | 8.0 |
| **General Health Comparison to One Year Ago** | | |  |  |  |
| *Much better than one year ago* | 9.9 | 14.0 | 8.3 | 4.8 | 10.0 |
| *Somewhat better than one year ago* | 7.1 | 11.0 | 6.2 | 17.0 | 9.6 |
| *About the same* | 23.0 | 57.0 | 49.0 | 27.0 | 40.0 |
| *Somewhat worse than one year ago* | 33.0 | 17.0 | 32.0 | 27.0 | 27.0 |
| *Much worse than one year ago* | 27.0 | 1.5 | 4.2 | 24.0 | 14.0 |
| **Activities Of Daily Living - ADL’s - % A Lot or A Little** | | |  |  |  |
| Trouble with Vigorous Activities | 96.0 | 36.0 | 58.0 | 81.0 | 67.0 |
| Trouble with Moderate Activities | 79.0 | 6.6 | 17.0 | 54.0 | 39.0 |
| Trouble Lifting | 81.0 | 8.0 | 20.0 | 49.0 | 40.0 |
| Trouble Climbing Several Stairs | 93.0 | 26.0 | 47.0 | 70.0 | 59.0 |
| Trouble Climbing One Stair | 77.0 | 9.5 | 17.0 | 51.0 | 39.0 |
| Trouble Bending | 77.0 | 13.0 | 26.0 | 56.0 | 43.0 |
| Trouble Walking Mile | 93.0 | 13.0 | 28.0 | 65.0 | 50.0 |
| Trouble Walking Several Blocks | 84.0 | 5.1 | 16.0 | 57.0 | 40.0 |
| Trouble Walking One Block | 59.0 | 1.5 | 6.2 | 24.0 | 24.0 |
| **Social Isolation and Loneliness Measures** | | |  |  |  |
| **Summary Lubben Social Network Scale (Median,IQR)** | 12 (9, 17) | 19 (15, 23) | 18 (12, 21) | 20 (14, 24) | 17 (11, 21) |
| **How many relatives do you see or hear from at least once a month** | | | | | |
| *None* | 4.3 | 2.2 | 2.1 | 0.0 | 2.5 |
| *One* | 7.8 | 2.9 | 4.2 | 1.6 | 4.6 |
| *two* | 12.0 | 8.8 | 8.3 | 11.0 | 10.0 |
| *3 or 4* | 30.0 | 19.0 | 36.0 | 22.0 | 27.0 |
| *5 to 8* | 28.0 | 32.0 | 29.0 | 30.0 | 30.0 |
| *9 or more* | 18.0 | 35.0 | 20.0 | 35.0 | 26.0 |
| **How many relatives – feel at ease with private matters** | | | | | |
| *None* | 13.0 | 2.9 | 4.2 | 4.8 | 6.9 |
| *One* | 17.0 | 8.0 | 9.4 | 3.2 | 11.0 |
| *two* | 27.0 | 19.0 | 20.0 | 17.0 | 22.0 |
| *3 or 4* | 22.0 | 28.0 | 35.0 | 22.0 | 27.0 |
| *5 to 8* | 13.0 | 23.0 | 24.0 | 30.0 | 21.0 |
| *9 or more* | 7.1 | 20.0 | 7.3 | 22.0 | 13.0 |
| **How many relatives - feel close to call on them for help** | | | | | |
| *None* | 11.0 | 1.5 | 3.1 | 0.0 | 4.8 |
| *One* | 14.0 | 8.8 | 9.4 | 4.8 | 10.0 |
| *two* | 32.0 | 15.0 | 22.0 | 21.0 | 23.0 |
| *3 or 4* | 18.0 | 27.0 | 36.0 | 25.0 | 26.0 |
| *5 to 8* | 16.0 | 28.0 | 22.0 | 27.0 | 22.0 |
| *9 or more* | 8.5 | 20.0 | 7.3 | 22.0 | 14.0 |
| **How many friends do you see or hear from at least once a month** | | | | | |
| *None* | 23.0 | 7.3 | 16.0 | 11.0 | 15.0 |
| *One* | 12.0 | 4.4 | 3.1 | 0.0 | 5.9 |
| *two* | 15.0 | 9.5 | 11.0 | 14.0 | 12.0 |
| *3 or 4* | 25.0 | 29.0 | 22.0 | 21.0 | 25.0 |
| *5 to 8* | 11.0 | 20.0 | 27.0 | 24.0 | 19.0 |
| *9 or more* | 13.0 | 30.0 | 21.0 | 30.0 | 23.0 |
| **How many friends ease with private matters** | | | | | |
| *None* | 40.0 | 17.0 | 19.0 | 21.0 | 25.0 |
| *One* | 20.0 | 10.0 | 16.0 | 6.3 | 14.0 |
| *two* | 16.0 | 18.0 | 18.0 | 17.0 | 17.0 |
| *3 or 4* | 14.0 | 27.0 | 18.0 | 19.0 | 20.0 |
| *5 to 8* | 4.3 | 14.0 | 21.0 | 17.0 | 13.0 |
| *9 or more* | 5.0 | 14.0 | 9.4 | 19.0 | 11.0 |
| **How many friends feel close to call on them for help** | | | | | |
| *None* | 38.0 | 12.0 | 19.0 | 19.0 | 23.0 |
| *One* | 21.0 | 12.0 | 9.4 | 9.5 | 14.0 |
| *two* | 16.0 | 18.0 | 24.0 | 16.0 | 18.0 |
| *3 or 4* | 17.0 | 25.0 | 20.0 | 19.0 | 20.0 |
| *5 to 8* | 3.5 | 17.0 | 22.0 | 22.0 | 14.0 |
| *9 or more* | 4.3 | 18.0 | 6.2 | 14.0 | 10.0 |
| **Total UCLA Loneliness Score** | 3.00 (0.00, 6.00) | 0.00 (0.00, 0.00) | 4.00 (3.00, 6.00) | 1.00 (0.00, 3.00) | 1.00 (0.00, 4.00) |
| **How often do you feel that you lack companionship?** | | | | | |
| *None* | 49.0 | 91.0 | 19.0 | 60.0 | 57.0 |
| *Hardly ever* | 11.0 | 6.6 | 24.0 | 19.0 | 14.0 |
| *some time* | 19.0 | 2.2 | 39.0 | 9.5 | 17.0 |
| *often* | 21.0 | 0.0 | 19.0 | 11.0 | 12.0 |
| **How often do you feel left out?** | | | | | |
| *None* | 52.0 | 97.0 | 21.0 | 59.0 | 60.0 |
| *Hardly ever* | 17.0 | 2.9 | 34.0 | 19.0 | 17.0 |
| *some time* | 16.0 | 0.0 | 31.0 | 11.0 | 14.0 |
| *often* | 16.0 | 0.0 | 14.0 | 11.0 | 9.6 |
| **How often do you feel isolated from others?** | | | | | |
| *None* | 45.0 | 96.0 | 12.0 | 62.0 | 57.0 |
| *Hardly ever* | 16.0 | 2.9 | 29.0 | 22.0 | 16.0 |
| *some time* | 19.0 | 0.7 | 42.0 | 3.2 | 16.0 |
| *often* | 20.0 | 0.0 | 17.0 | 13.0 | 12.0 |

Supplementary Table 2 – Baseline Characteristics and Complications Related to COVID-19 Hospitalization (March – May 2020)

| **Characteristic** | **Cluster 0** (141) | **Cluster 1** (137) | **Cluster 2** (96) | **Cluster 3** (63) | **Total** (437) |
| --- | --- | --- | --- | --- | --- |
| **BASELINE HEALTH CHARACTERISTICS AND COMORBIDITIES** | | | | |  |
| Healthcare worker | 5.7 | 8.0 | 7.3 | 4.8 | 6.6 |
| Number of medications  (Median, IQR) | 5.0 (2.0, 9.0) | 1.0 (0.0, 3.0) | 3.0 (1.0, 6.2) | 4.0 (1.5, 7.0) | 3.0 (1.0, 6.0) |
| Obesity (BMI ≥ 30) | 35.0 | 24.0 | 28.0 | 48.0 | 32.0 |
| Active cancer* | 2.8 | 2.9 | 4.2 | 4.8 | 3.4 |
| Diabetes | 32.0 | 24.0 | 30.0 | 41.0 | 30.0 |
| Stroke | 14.0 | 2.2 | 4.2 | 3.2 | 6.6 |
| Heart failure | 9.2 | 2.9 | 3.1 | 4.8 | 5.3 |
| ARDS | 2.1 | 2.9 | 3.1 | 97.0 | 16.0 |
| CAD | 16.0 | 6.6 | 6.2 | 14.0 | 11.0 |
| Hypertension | 67.0 | 44.0 | 51.0 | 56.0 | 54.0 |
| Renal disease | 20.0 | 4.4 | 2.1 | 9.5 | 9.6 |
| COPD | 5.0 | 0.7 | 2.1 | 7.9 | 3.4 |
| Asthma | 11.0 | 4.4 | 11.0 | 13.0 | 9.2 |
| Interstitial lung disease | 0.0 | 0.0 | 1.0 | 1.6 | 0.5 |
| Sleep apnea | 7.8 | 0.7 | 6.2 | 11.0 | 5.7 |
| Other lung disease | 5.0 | 1.5 | 2.1 | 1.6 | 2.7 |
| HIV | 3.5 | 0.7 | 1.0 | 3.2 | 2.1 |
| IBD | 0.0 | 0.0 | 1.0 | 0.0 | 0.2 |
| Rheumatic disease or immunosuppressed state** | 7.8 | 3.6 | 6.2 | 9.5 | 6.4 |
| Current or past smoker | 27.0 | 18.0 | 19.0 | 29.0 | 22.0 |
| **IN-HOSPITAL COMPLICATIONS/ MEDICATIONS** | | | | | |
| Length of Hospital Stay (Median, IQR) | 4 (3, 10) | 4 (2, 7) | 4 (2, 8) | 40 (24, 67) | 5 (3, 12) |
| Had Hypoxia at ED | 44.0 | 41.0 | 38.0 | 78.0 | 46.0 |
| Intubated | 1.4 | 0.7 | 0.0 | 100.0 | 15.0 |
| Time Intubated  (Median, IQR) | 0 (0, 0) | 0 (0, 0) | 0 (0, 0) | 35 (20, 65) | 0 (0, 0) |
| Acute Kidney Injury | 18.0 | 8.8 | 14.0 | 63.0 | 21.0 |
| Heart Failure | 1.4 | 0.7 | 1.0 | 11.0 | 2.5 |
| Septic Shock | 0.7 | 2.2 | 0.0 | 25.0 | 4.6 |
| Ventilator related Pneumonia | 0.0 | 0.0 | 0.0 | 33.0 | 4.8 |
| Myocardial Infarction | 2.8 | 2.2 | 0.0 | 4.8 | 2.3 |
| New onset Arrythmia | 5.7 | 2.9 | 1.0 | 25.0 | 6.6 |
| Rhabdomyolysis | 0.7 | 1.5 | 0.0 | 6.3 | 1.6 |
| Other complications | 23.0 | 11.0 | 18.0 | 68.0 | 24.0 |
| Dialysis | 9.2 | 0.7 | 0.0 | 25.0 | 6.9 |
| VTE | 5.0 | 3.6 | 4.2 | 37.0 | 8.9 |
| Use of Steroids | 13.0 | 5.1 | 11.0 | 75.0 | 19.0 |
| New onset Cardiac disease, Hypertension, Diabetes or Heart Failure | 13.0 | 15.0 | 3.1 | 17.0 | 12.0 |

Abbreviations: CKD = Chronic Kidney Disease/Creatinine >2 mg/dL on admission. COPD = Chronic Obstructive Pulmonary Disease. HIV = Human Immunodeficiency Virus. ARDS = Acute Respiratory Distress Syndrome. IBD = Inflammatory Bowel Disease. VTE = Venous Thromboembolism.

*Cancer: excluding non-melanoma skin cancer.

**Other immunosuppressed state: Chemotherapy or radiotherapy within last 6 months, or inherited immunodeficiency.
